# Linking dietary intake, circadian biomarkers, and clock genes on obesity: A study protocol

**DOI:** 10.3389/fnut.2023.1134789

**Published:** 2023-04-11

**Authors:** Marlene Lages, Renata Barros, Sara Carmo-Silva, Maria P. Guarino

**Affiliations:** ^1^ciTechCare—Center for Innovative Care and Health Technology, Polytechnic of Leiria, Leiria, Portugal; ^2^Faculty of Nutrition and Food Science, University of Porto, Porto, Portugal; ^3^Laboratory for Integrative and Translational Research in Population Health (ITR), University of Porto, Porto, Portugal; ^4^EPIUnit—Institute of Public Health, University of Porto, Porto, Portugal; ^5^Polytechnic Institute of Castelo Branco, Castelo Branco, Portugal; ^6^ESSLei, School of Health Sciences, Polytechnic of Leiria, Leiria, Portugal

**Keywords:** obesity, circadian rhythms, chrononutrition, nutrigenetics, genes, biomarkers, metabolism

## Abstract

**Background:**

The prevalence of obesity continues to rise, and although this is a complex disease, the screening is made simply with the value of the Body Mass Index. This index only considers weight and height, being limited in portraying the multiple existing obesity phenotypes. The characterization of the chronotype and circadian system as an innovative phenotype of a patient’s form of obesity is gaining increasing importance for the development of novel and pinpointed nutritional interventions.

**Objective:**

The present study is a prospective observational controlled study conducted in Portugal, aiming to characterize the chronotype and determine its relation to the phenotype and dietary patterns of patients with obesity and healthy participants.

**Methods:**

Adults with obesity (study group) and healthy adults (control group), aged between 18 and 75, will be enrolled in this study. Data will be collected to characterize the chronotype, dietary intake, and sleep quality through validated questionnaires. Body composition will also be assessed, and blood samples will be collected to quantify circadian and metabolic biomarkers.

**Discussion:**

This study is expected to contribute to a better understanding of the impact of obesity and dietary intake on circadian biomarkers and, therefore, increase scientific evidence to help future therapeutic interventions based on chronobiology, with a particular focus on nutritional interventions.

## 1. Introduction

The prevalence of obesity continues to rise, predicted to become the biggest epidemic in history. According to the World Health Organization, around 2 billion adults are overweight; 50 million have obesity. Most of the world’s population lives in countries where obesity and overweight kill more people than underweight (1). Obesity is characterized by an excess of body fat, which can be measured by body composition analysis. Nevertheless, clinical obesity is classified based on the body mass index (BMI), expressed as the ratio of body weight in kilograms divided by the height in square meters (2, 3). Large population studies described the relationship between a higher BMI and increased mortality/morbidity risk (2, 4, 5). The BMI has been used to stratify patients into risk categories and to monitor changes in adiposity since it is an easy, affordable, and quick tool for clinical use, despite its limitations (6, 7).

Different obesity phenotypes are associated with additional cardiovascular risk (8, 9). Some of the phenotypes described in the literature are Metabolically Healthy Obesity, in which a high BMI is associated with an apparently healthy metabolic profile, lower visceral adipose tissue, high values of lean mass, and high cardiorespiratory fitness. This phenotype may transition to metabolically unhealthy obesity and is associated with an increased risk of cardiovascular diseases. Another phenotype is described as Metabolic Obesity with Normal Weight, in which individuals, despite having normal weight, present high values of visceral adipose tissue, lower insulin sensitivity, hyperinsulinemia, dyslipidemia, and increased plasma levels of pro-inflammatory cytokines. Normal weight obesity syndrome presents values of body fat mass above 30% with BMI values within the healthy range. Lastly, there is the Sarcopenic Obesity phenotype, characterized by low skeletal mass and increased fat mass percentage (10). Considering these different phenotypes, the BMI classification is insufficient to adequately stratify patients with obesity regarding types of adiposity, cardiovascular risk or adequacy of therapeutic interventions. In addition to the BMI, clinical practice guidelines suggest associating the measurement of the waist circumference as a reference for abdominal fat (11, 12), however, this measure does not differentiate subcutaneous from visceral fat deposition.

The knowledge regarding the etiology of obesity is still evolving; however, some gaps remain, since the translation of this knowledge into the treatment and management of this disease is yet to be successful in large-scale clinical programs (13). Obesity is a complex condition, therefore not to be considered a homogenous state, considering the noticeable heterogeneity observed among people that meet the current medical diagnostic criteria for obesity (BMI ≥ 30 kg/m^2^).

Obesity classes I and II encompass different health risks associated with factors that include body fat distribution, overall nutritional quality, physical activity levels, and cardiorespiratory fitness (14). Some people, although they have obesity, eat a nutritionally balanced diet and are physically active (15). Other individuals only show moderate obesity, however, when accompanied by visceral obesity, they can exhibit features of metabolic syndrome (16). These remarks underline the idea that BMI alone might not be sufficient to assess health risks, characterize, and diagnose obesity. Piché and colleagues (2) recently proposed that the singular term “obesity” does not properly define the diverse types of obesities, regarding adipose tissue type and function, body fat distribution, and patient lifestyle habits. These authors suggest that the concept of different “obesities” is more adequate to the clinical reality and to the distinct treatment challenges associated with different categories of obesity.

Current evidence indicates that there is a reciprocal interaction between metabolism and the circadian system. Primarily, a comprehensive neural network connects the suprachiasmatic nucleus (SCN) of the hypothalamus to the energetic centers implicated in, for example, food intake, sleep/wakefulness and energy expenditure (17). The effect of the circadian system on metabolism, mediated by the SCN and peripheral clocks, is reflected in the circadian fluctuations exhibited by several metabolically significant hormones (17, 18). Changes in hormonal rhythms might lead to internal malfunction and are related to metabolic dysfunctions that predispose the development of metabolic diseases such as obesity (18–22). While the SCN is mainly synchronized by light/dark cycles, peripheral clocks are entrained by feeding/fasting cycles (23, 24). Besides meal timing, nutrients can also induce phase shifts in the peripheral circadian clock. Hirao and colleagues found that a combination of carbohydrates and protein is necessary to change the mouse liver clocks (25). As reviewed by Oike and colleagues (26), nutrients such as glucose and amino acids quickly alter the expression of clock genes, particularly Per2 and Rev-erbα (27–30). Skipping breakfast or having a lower food intake in the first meal of the day alongside with high-caloric dinners impairs peripheral clock gene expression and results in higher daily glucose excursions in animals (31–33). A study conducted by Jakubowicz and colleagues on individuals with type 2 diabetes showed that calories consumed at breakfast or dinner affected the daily rhythm of postprandial glycaemic excursion and insulin levels (34). These authors also reported that skipping breakfast negatively affected clock and clock-controlled gene expression (35).

Despite the contribution in elucidating important mechanisms in chronobiology, the majority of the studies do not account for differences of circadian rhythmicity among individuals. Distinct chronotypes reflect different timings of circadian behavior, physiology, and even patterns of clock genes expression. Besides regulating sleep, the chronotype can have a major influence on appetite. Thus, the characterization of individual chronotypes (expression of circadian rhythmicity), as part of a patient phenotype, is gaining increasing importance for the development of novel and pinpointed nutritional interventions. A randomized clinical trial showed that a chronotype-adjusted diet was more effective than the traditional hypocaloric diet in the improvement of anthropometrical parameters in patients with obesity (36). Individual circadian variations may be valuable, and a critical first step, for providing information to design therapeutic strategies and to help “fine-tune” chronobiology interventions, including chrononutrition approaches (37). Research in this field is currently hindered by the fact that only a few studies assessed simultaneously the chronotype and nutrient intake and timing during the interventions. Furthermore, there is no standard method to objectively define chronotypes. Usually, this is determined by a combination of questionnaires, evaluation of clock genes expression and/or protein levels, determination of non-invasive clock outputs, such as core body temperature, and heterogeneous pools of biomarkers that act as readouts of the intrinsic circadian rhythms (38–41). Although there is an increasing interest in chronotype, as a novel dimension of nutrition and health, there is still a lack of consistent evidence regarding its relation to obesity and metabolic disturbances (42, 43). Researchers in this field highlight the need for more research and proper characterization of clock gene expression and circadian biomarkers in obesity. Furthermore, most studies have a high risk of bias and do not evaluate parameters related to metabolic dysfunction such as adiponectin, leptin, and insulin (42, 44).

The relationship between circadian rhythm disruption and obesity is complex and not fully understood. More studies are necessary to assess the role of meal schedules and dietary composition in the regulation and/or deregulation of peripheral clocks, especially including the biological differences in the circadian system through the assessment of individual chronotypes.

The present study aims to conduct an observational study on chronotype, and its relation to phenotype and dietary intake in patients with obesity, compared with healthy individuals. This study will contribute with scientific evidence to help future interventions based on chronobiology, specially chrononutrition.

## 2. Methods and analysis

### 2.1. Study design

NutriClock is a prospective observational study involving adults with obesity (study group) and healthy adults (control group). The study protocol was developed based on the Standard Protocol Items: Recommendations for Interventional Trials (SPIRIT) guidelines (45).

### 2.2. Participant selection

Participants will be recruited from healthcare centers in Leiria, Portugal. The study will also be disseminated through social networks and institutional emails, in an attempt to reach more participants.

The eligibility of the participants will be assessed on the first visit by the assessment of inclusion and exclusion criteria after the potential participants signed the informed consent. The observational study will be conducted on adults and recruitment will be gender-independent. Obesity will be defined according to the criteria of the World Health Organization (1). The detailed participant inclusion and exclusion criteria are listed in Table 1.

**Table 1:** Inclusion and exclusion criteria for the participants in both study groups.

**Inclusion criteria**
Adults aged ≥ 18 and ≤ 75 years. BMI ≥ 30 kg/m^2^ (study group). BMI ≥ 18.5 and < 25.0 kg/m^2^ (control group).
**Exclusion criteria**
Individuals unwilling or unable to give their informed consent. Severe psychiatric conditions and/or inability to understand and engage in the study. Night or rotating shift workers, or individuals that crossed more than two times zones in the 2 weeks before the beginning of the study. Diagnosed sleep disorders, including severe Obstructive Sleep Apnea. Pregnant women^1^. Individuals with an electronic medical device/implant^1^. Infection 4 weeks before baseline assessment. Regular use (equal to or greater than weekly) of the following medications: Wakefulness-promoting agents modafinil, amphetamine derivates, methylphenidate. Sedatives including benzodiazepines, Z-drugs (zopiclone, zolpidem and zaleplon). Melatonin, including circadian and melatonin analogs. Clonazepam and other drugs for nocturnal movement disorders. Probiotics. Anti-obesity drugs (orlistat, liraglutide). Glucocorticoids.

1 Assessment of body composition with the bioelectrical impedance analysis (BIA) equipment is not possible in these conditions.

### 2.3. Sample size

The sample size was estimated using the following formula (46):


$$n\;\left(eachgroup\right)=\frac{\left(p_{0}q_{0}+p_{1}q_{1}\right){\left(z_{1-\alpha /2}+z_{1-\beta }\right)}^{2}}{{\left(p_{1}-p_{0}\right)}^{2}}$$

- α is the value of alpha, conventionally 0.05 (two-sided).
- β is the value of beta, conventionally 0.20.
- p_0_ is the expected proportion of controls with exposure.
- p_1_ is the proportion of cases with exposure.
- q_0_ = (1 – p_0_).
- q_1_ = (1 – p_1_).

The prevalence of obesity in the Portuguese adult population was reported as being 17.7% in 2019 (47). Strong evidence suggest that circadian misalignment contributes to obesity and that the metabolic disorders’ genetic components underlie the large interindividual variation in body weight. Studies have highlighted that the genetic component can contribute for 40%–70% of obesity cases (48). Considering that the chronotype is related to clock gene expression, we assumed that an average of 55% of individuals with obesity will have a clock gene-mediated genetic contribution to the obesity phenotype. Based on these assumptions:

- p_0_ = proportion of controls with obesity = 0.18.
- p_1_ = proportion of obesity cases with chronotype misalignment = 0.55.
- q_0_ = (1 – p_0_) = 1–0.18 = 0.82.
- q_1_ = (1 – p_1_) = 1–0.55 = 0.45.
- z _(1 – α/2)_ = 1.96 (value of the standard normal distribution corresponding to a significance level of alpha [1.96 for a 2-sided test at the 0.05 level]).
- z _(1 – β)_ = 0.84 (value of the standard normal distribution corresponding to the desired level of power −80%).

The sample size necessary for this study is of 46 participants (23 cases and 23 controls). Having into consideration an attrition rate of 40% for visits 2 and 3, the minimum sample size estimated is 64 (32 for each group), to prevent and account for the potential high drop-out rate, incomplete data, and consent withdrawal.

### 2.4. Data collection timeline

Data collection is expected to be conducted from January 2023 to September 2023, though, this period may be extended to ensure a sufficient sample number, if necessary. Once recruitment begins, all potential participants will be invited to enter the observational study. The study aims and procedures will be explained in detail and in a simple and understandable manner to the potential participants, so that they can make an informed decision, on whether to accept or decline to participate. When a potential participant agrees to join the study, they are required to provide written informed consent. Only after this initial step will data be collected to assess the eligibility of participants.

**Figure 1:**
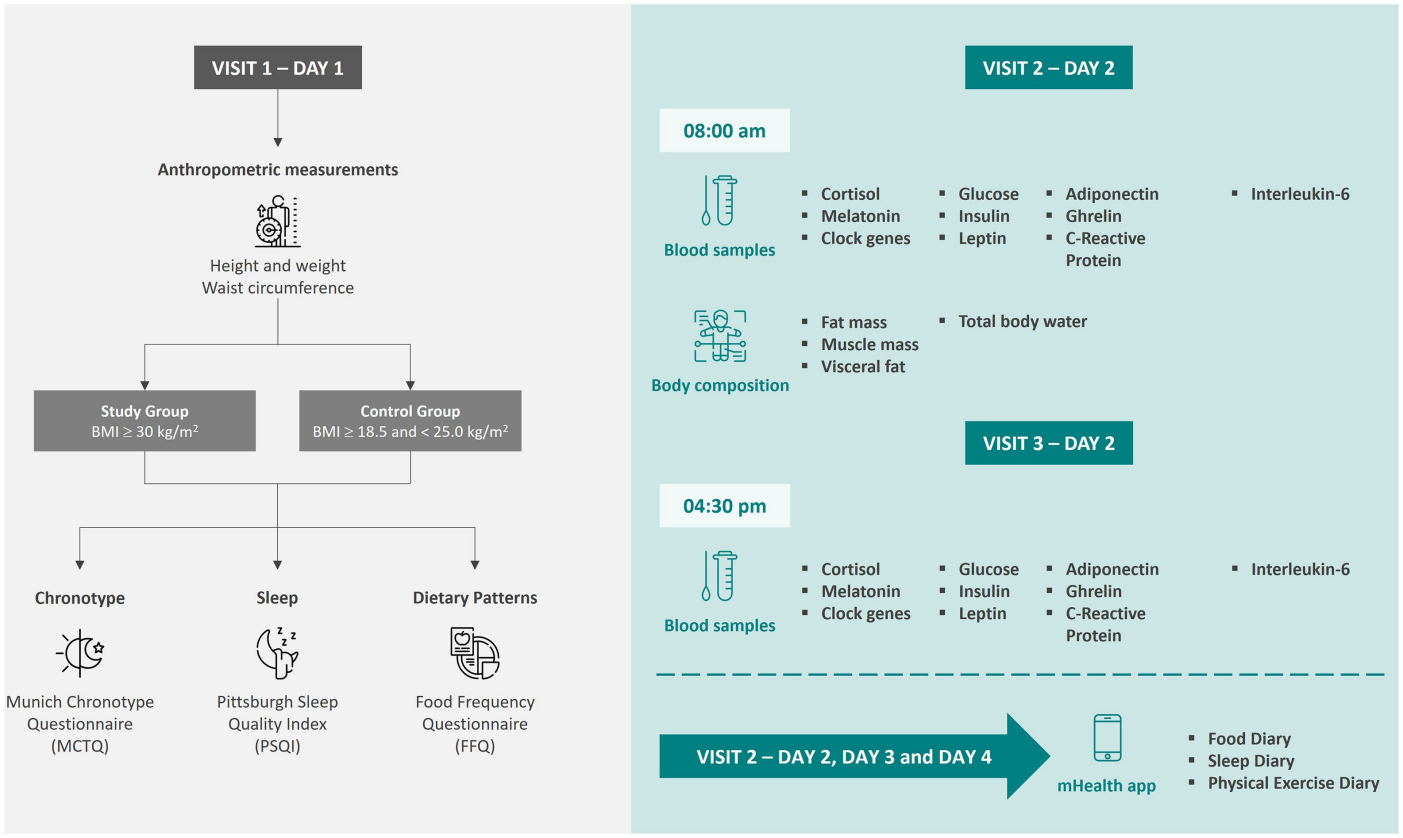
Data collection time points and sample collections for the participants of the observational study. Icons made by Freepik from www.flaticon.com. Icons made by iconixar from www.flaticon.com. Icons made by Sweetline from www.flaticon.com.

Data will be collected from eligible adults in three visits, each one with an expected duration of about 1 h. Figure 1 summarizes the study design, including the data and samples to be collected at each time point of the observational study.

### 2.5. Measurements

#### 2.5.1. Anthropometric and body composition assessments

Participants’ body weight, height, and waist circumference will be measured according to the Portuguese Directorate General of Health guidelines for anthropometric procedures in adults (49). Body mass index (BMI; kg/m^2^) will be calculated and categorized according to the age and sex-specific BMI cut-offs for adults (50). Fat mass (FM), visceral adipose tissue (VAT), fat-free mass (FFM), and skeletal muscle mass (SMM) for arms, legs, torso, and whole body will be assessed using the bioimpedance equipment seca mBCA 525 (Bacelar, Porto, Portugal).

#### 2.5.2. Biochemical assessments

Peripheral blood samples (15–20 mL) will be collected from the participants at 08:00 a.m., after overnight fasting, and 04:30 p.m. on the same day, and aliquoted into EDTA tubes following safety standards, by certified healthcare professionals. Another blood sample (2.5 mL) will be collected from volunteers to PAXgene® Blood RNA Tubes (BD Biosciences), to obtain total RNA from whole blood.

For the total RNA from peripheral blood mononuclear cells (PBMC), blood samples will be diluted in 25 mL of Phosphate Buffered Saline (PBS) 1 × and added to 50 mL falcon tubes with 10 mL of Histopaque, density 1.077 g/mL. Samples will be centrifugated at 20^°^C to 22^°^Cand plasma will be immediately aliquoted and stored at −80^°^C. The interface bands, which contain the PBMC, will be aspirated into new 50 ml falcon tubes and washed with PBS 1 ×, followed by centrifugation at 21^°^C–25^°^C. Supernatants will be discarded and PBMC aliquots will be stored at −80^°^C until the analysis.

For the total RNA from whole blood, PAXgene® Blood RNA tubes will be centrifuged at 20 to 22°C and the supernatant removed. To the pellet, RNase-free water will be added and the samples vortexed until the pellet dissolution, followed by a centrifugation at 20^°^C to 22^°^C. After removal of the supernatant, a lysis buffer will be added, the sample vortexed and transferred to a 1.5 mL microcentrifuge tube where 40 μL of proteinase K will be added. After incubation, the sample will be transferred to a spin column of the Macherey Nagel RNA extraction kit, according to the manufacturer’s instructions. RNA concentration and purity will be determined using a spectrophotometer. Samples will be stored at −80^°^C until the analysis.

RNA samples will then be converted into cDNA and the mRNA levels of the clock genes (BMAL1, CLOCK, PER1-3, CRY1-2, CSNK1ε, REV-ERBα, REV-ERBβ, and DEC1) will be assessed using real-time quantitative reverse transcriptase polymerase chain reaction (qRT-PCR). Relative gene expression will be calculated according to the ΔCT method (51, 52).

Plasma levels of insulin, leptin, adiponectin, ghrelin, c-reactive protein, and interleukin-6 will be quantified using ELISA kits, according to the manufacturer’s instructions. Glucose will be assessed using the hexokinase method with a commercial kit.

#### 2.5.3. Clinical data

Participants will register in the NutriClock mobile application, specifically designed for this observational study by a multidisciplinary team, including computer engineers, nutritionists, physiologists, and psychologists (53). The NutriClock mobile application and backoffice were designed and developed to comply with ethical standards and the General Data Protection Regulation. The research team and authorized healthcare professionals will have access to the information that each participant introduces in the mobile application *via* the backoffice platform.

The clinical history information may be entered voluntarily by the participant when creating the account. Participants can also add or edit information in their profile after they are registered, at any time point. Healthcare professionals can also complete the information about the participant in the NutriClock backoffice. The information that will be collected includes sociodemographic data, diagnosed pathologies, and current medication.

#### 2.5.4. Dietary intake assessments

Dietary intake will be assessed using two methodologies: a 3-day food diary included in the NutriClock app (53) and a Food Frequency Questionnaire (FFQ). To ensure equal access and opportunity to participate in the research study, regardless of the socioeconomic level, availability of a smartphone, and/or ability to use it, participants can opt to complete the 3-day food diary in the traditional pen-and-paper format.

The participants will start filling out the 3-day food diary on the day of their visit to the research center to collect biological samples (Visit 2, Figure 1). All foods, including drinks, beverages, and dietary supplements consumed during the 3 days should be registered. Participants will also be required to register the meal time and type (breakfast, lunch, dinner or snack). Food portions will be estimated using either a photographic method (participants can take a picture of their meal through the mobile application), using the weight or volume, pre-determined household measures, and/or default mean portions where the participants cannot quantify a specific food item.

The conversion of food into energy and nutrients will be based on the Portuguese Food Composition Table (54), incorporated into the app and, if necessary, other food composition tables. In the app’s food diary section, participants can also upload pictures of the nutritional information/food labeling of the food items being consumed, if available. In the backoffice, it is possible to continuously adapt and complete the information on the Portuguese Food Composition Table by adding nutritional information for new food items or meals. The Portuguese Food Frequency Questionnaire (FFQ) is a validated semi-quantitative instrument that assesses dietary intake, reporting the previous 12 months. This tool will be used to collect information on the consumption of less frequently eaten foods and, therefore, minimize interferences with the day-to-day variation of food intake (55). The Portuguese FFQ includes a pre-determined list of 86 food items or groups, and nine frequency categories ranging from “Never or less < 1 per month” to “6+ per day” (56). Food consumption is quantified by multiplying the reported intake frequency by the food item or group average standard portion, and by a seasonal variation factor for items consumed only in a specific season. The conversion to nutrients will be supported by a compilation of databases on the nutritional food composition, including the Portuguese Food Composition Table (54).

The data collected with the FFQ will be mainly used to perform a global characterization of the participants’ dietary intake in the previous 12 months. The data collected using the 3-day food diary will be used to correlate dietary intake and circadian biomarkers.

#### 2.5.5. Questionnaires

To complete the participant’s characterization and data, a set of validated questionnaires and instruments will be used.

The Pittsburgh Sleep Quality Index (PSQI) is a 19-items (plus 5 additional items, in case the responder has a roommate or bed partner, that do not contribute to the total score) self-reported questionnaire, designed to subjectively assess global sleep quality based on the participant’s retrospective evaluation (considers the usual sleep habits over the past one-month period). The items of the PSQI are divided into seven components, namely, subjective sleep quality, sleep latency, sleep duration, habitual sleep efficiency, sleep disturbances, use of sleeping medication, and daytime dysfunction, each weighted equally on a scale from 0 to 3. The global PSQI score ranges from 0 to 21, corresponding to the sum of the seven components. A global PSQI score greater than 5 indicates major difficulties in at least two components or moderate difficulties in more than three components (57, 58). This instrument has been validated for the Portuguese population (59).

The Munich Chronotype Questionnaire (MCTQ) was developed by Roenneberg and colleagues to assess the circadian phase of entrainment, which is considered a quantifiable biological phenotype (41, 60). The questions aim to evaluate sleep behavior on workdays and work-free days, separately, since this distinction is essential to determine the participant’s chronotype. The chronotype resultant from the MCTQ is expressed in local time and it is based on the Mid-Sleep time in Free days (MSF) after correction for potential compensatory sleep (MSF_sc_) (60). The midpoint of sleep has been considered one of the most accurate behavioral markers to determine the circadian phase (60, 61). However, to account for the sleep debt as a cofounder, it is necessary to calculate the average sleep duration of the entire week and correct MSF by subtracting half of the oversleep. According to the results of the MCTQ database, Roenneberg and colleagues identified seven groups based on MSF_sc_, namely, extremely early, moderately early, slightly early, intermediate, slightly late, moderately late and extremely late (60). This instrument has already been validated for the Portuguese population (62).

### 2.6. Statistical analysis

Statistical analysis will be performed using the IBM SPSS Statistics software version 28.0.1. (IBM Corp., Warrington, United Kingdom) and GraphPad Prism version 8 (GraphPad Software, Inc.; San Diego, United States). Continuous variables will be presented as the mean ± standard deviation (SD), median, and interquartile range [Q1–Q3]. Categorical variables will be presented in frequency and percentage. The normality of continuous variables will be tested using the Shapiro–Wilk test if *n* < 50 or the Kolmogorov–Smirnov test if *n* ≥ 50. Parametric tests (Independent Sample *t*-test) will be used to compare the differences between the study and the control groups if the data has a normal distribution, otherwise, non-parametric tests (Mann–Whitney *U*-tests) will be performed. According to the normality or non-normality of data distribution, Spearman’s rank-order correlation (ρ) or Pearson’s correlation coefficient (r) will be performed to calculate and assess the strength of correlations between the study variables. Statistical significance will be set at *p* < 0.05.

## 3. Discussion

In recent decades, there have been breakthroughs in obesity care and treatment. However, despite these advances, there is a major remaining challenge: improving the understanding of the heterogeneity of obesity. This knowledge is essential to determine the best approaches to screen, assess risk, and select personalized and individualized treatments. The findings of this study will provide a better understanding of obesity phenotypes in regard to their chronotype, dietary intake (including meal time and nutrient intake), and circadian and metabolic biomarkers.

The circadian system regulates metabolism through the secretion of hormones, such as cortisol and melatonin (63). Alterations in melatonin and cortisol rhythms, or their blunted secretion, are important attributes of circadian misalignment (64, 65). Melatonin is a major regulator of circadian rhythm, namely sleep–wake cycles and hormone secretion (66). The levels of this hormone typically rise at night and fall during the day, in synchrony with the body’s circadian rhythm. Studies have shown that melatonin can help regulate glucose homeostasis by improving insulin sensitivity and reducing insulin resistance (67, 68). In addition, melatonin has been shown to improve lipid metabolism by reducing the levels of triglycerides and cholesterol in the blood. It may also play a role in regulating appetite and body weight (68–70). Disruptions to the body’s circadian rhythm can lead to alterations in melatonin secretion patterns which can have negative effects on metabolism and overall health, as disrupted melatonin secretion has been associated with an increased risk of metabolic disorders such as type 2 diabetes and obesity. Cortisol is tightly regulated by the circadian system, showing an anti-phasic pattern of secretion compared to melatonin (71). Disturbances in the normal circadian patterns can promote alterations in cortisol secretion patterns, which might have a deleterious effect on metabolism. Cortisol is a catabolic hormone with several essential functions in the body, including the mobilization of glucose from glycogen, amino acids and fats in response to other hormonal signals (71). Prolonged cortisol release can promote insulin resistance and obesity can promote abnormal cortisol levels (66).

Circadian oscillations of gene expression also regulate whole-body metabolism (72). For example, Gaspar et al. showed that patients with obstructive sleep apnea, which is strongly related to obesity (73, 74), have different patterns of clock gene expression compared with healthy individuals (51), further highlighting the importance of assessing the circadian features in disease.

The importance of determining the circadian rhythm has been extensively reported; although it started more focused on pharmacological studies (40), this relevance has extended to nutrition. From this, the concept of chrononutrition emerged, mostly because peripheral circadian clocks are mainly synchronized by food intake (24, 31). Furthermore, the circadian clock also regulates nutrient challenges, through transcription factors that modulate the expression of genes with regulatory roles in nutrient pathways (37, 75) Therefore, there is the potential to improve metabolic homeostasis by coordinating behavioral changes with the body’s daily rhythm. The identification of genetic patterns and signatures in response to dietary intake may guide the advance of new therapeutic approaches.

Designing dietary interventions that account for the quality, quantity and timing of food intake could be an approach to modulate the circadian rhythm and clock-controlled genes and optimize the synchronization of central and peripheral clocks, with a positive impact on overall metabolism. Therefore, the characterization of circadian and dietary makers is a critical first step to underpin therapeutic strategies and to support the adjustment of chrononutritional approaches for the management of chronic diseases such as obesity. In the present study, caloric intake, macronutrients (proteins, carbohydrates, and lipids), and meal times (time of the first meal of the day, time of last meal of the day and overnight fasting period) will be evaluated, in order to assess the impact of dietary intake (timing and nutrients) on circadian rhythm markers.

The research protocol has the advantage of simultaneously evaluating dietary intake, circadian rhythm biomarkers, and metabolic biomarkers in blood samples, of both individuals with obesity and healthy individuals. Taking into account circadian rhythm dysfunction in obesity, a better understatement of the circadian clock might provide important clues regarding overall metabolism and disease progression, helping to design more fitting treatment approaches. Based on previous studies, we expect to find differences in the expression of circadian clock genes between the two groups, particularly, in the expression of BMAL1, PER2, CRY2, and REV-ERBα (51, 76–79).

To minimize the number of visits by participants to the study center, and to attempt to increase study adherence, the food diary, sleep diary, and physical exercise registry will be filled out through the mobile application NutriClock. Previous studies have shown increased satisfaction and preference for mobile applications for reporting dietary intake, compared with conventional methods (80), which is a particularly important observation supporting the methodology of our study. Also, as the data is collected through the NutriClock mobile application, it is immediately available to the researchers, preventing the participant from having to go to the study center to deliver the forms in paper format. Furthermore, if data is not being inputted into the mobile application, the participants receive notifications with reminders of the importance of the diaries. In the backoffice, the researcher is also able to detect immediately, through a color system, if the participants are not collecting data and can contact them, if necessary. The records inserted by participants are evaluated on a daily basis, and the system assigns one of four color levels to each component completed, such as the food or sleep diary. This evaluation is based on two criteria: the time elapsed since the start of registration and the number of registrations made. If the number of registrations exceeds 75% of the total days, the component is marked with a green tab. If the number is between 50% and 75%, the tab is yellow, while an orange tab indicates a number between 25% and 50%. A red tab is assigned if the number of registrations is less than 25%.

Some limitations of this study should be considered, including a potential low acceptance of the protocol, causing difficulties in recruitment, and a high drop-out rate. Therefore, a percentage of 20% of drop-out was taken into consideration when calculating the sample size. This anticipated difficulty in recruitment and acceptance of the study protocol is mostly related to the multiple sample collections, and the requirement of filling out a 3-day food diary, which could potentially be a burden for some participants. Initially, recruitment will be promoted in the Leiria (Portugal) area but may be extended to other areas of the central region of Portugal if necessary. Recruiting participants only in the Leiria area, although more convenient for logistical reasons, could also be considered a limitation, given that all study participants will represent the same geographical area and, therefore, we must be cautious in interpreting and extrapolating data. In addition, to mitigate drop-out, participants who complete the observational study will have the opportunity to have a nutrition education session with a registered nutritionist. Also, participants will have permanent access to all the data that they will introduce in the mobile application. Another potential limitation is related to social bias in the self-reporting questionnaires, especially, in the food diaries, which could lead to an underestimation of food intake (81, 82). The assessment of clock gene expression only at two-time points during the day also represents a pitfall of the study. The circadian rhythm integrity has been previously evaluated by looking at clock gene expression at four-time points in hospitalized patients, allowing a more robust interpretation of the results (50). This protocol is based on outpatient recruitment and collection of blood samples at two different time points during the day for outpatient volunteers carries a significant risk of increasing attrition of the study. To mitigate the risk of misinterpreting the data on circadian rhythm based on two-time point serum biomarkers and clock gene assessment, the Pittsburgh Sleep Quality Index and the Munich Chronotype Questionnaire were added to the protocol to broaden the information obtained by serological analysis.

In conclusion, this study will characterize and compare circadian rhythm biomarkers of healthy individuals and people with obesity, associating this information with dietary intake. The results of this study might provide new evidence to develop more targeted and personalized interventions for nutritionists, endocrinologists, and other healthcare professionals, for the treatment and prevention of obesity and its associated complications.

## 4. Ethics and dissemination

This is a research project that falls under the scope of Portuguese Law no. 21/2014 April 16 that covers all research with humans, including observational studies. This study will be carried out on adults over the age of 18 years. Adults who are unable to provide their informed consent will not be included in the study.

The research project was approved by the Ethics Committee of the Centre’s Regional Health Administration (ARS Centro) on 29th January 2021 (Proc no. 67-2020). The research will be carried out in strict compliance with the ethical principles of the Helsinki Declaration and obeying international, European, and national legislation, including the General Data Protection Regulation (Regulation (EU) 2016/679). The principles of autonomy, nonmaleficence and beneficence, fairness, and clear informed consent will be strictly respected. The researcher will assure that the informed consent document will be available to the volunteers before starting any procedure of the study. The recruitment will be gender-independent to minimize gender bias.

Biological samples will be protected by the principle of non-commercialization, commercial use, patenting, or any financial gain, and the principle of the non-susceptibility of patenting human genetic heritage. The samples will be used exclusively to support this research initiative and will be destroyed immediately after use.

The participants will not be identified by any of their data, to ensure the confidentiality and anonymity of the collected data, a unique ID number will be assigned to each participant. Only the Principal Investigator will have access to the list with the match between the participant’s ID and their data. This list will be in electronic format, with restricted access to its content and secured with a password, preventing confidential information from being accessed, printed, forwarded, or copied by an unauthorized person(s). Personal data collected in the context of this study, and shared between team members, will be kept in secure files, with restricted access, be they electronic or physical, and kept after the conclusion of this study, for the time defined by law.

Participants have the right to withdraw from the study at any time, without the need to provide a reason, if they wish not to do so. Participants will be assured that if they wish to withdraw from the study, their medical care will not be affected. Except if the participant indicates otherwise, any data collected up to the point of withdrawing consent will be included in the final analysis.

The results will be disseminated in group data, and individual results that might identify the participant will never be shown to the public. The results and deliverables of this research project will be available in academic health-related publications and through presentations in international scientific meetings.

## Data availability statement

The original contributions presented in the study are included in the article/supplementary material, further inquiries can be directed to the corresponding author.

## Ethics statement

The studies involving human participants were reviewed and approved by the Ethics Committee of the Centre’s Regional Health Administration. The patients/participants provided their written informed consent to participate in this study.

## Author contributions

ML, RB, SC-S, and MG conceived and designed the study, contributed to the protocol development, and contributed to the manuscript by revising it critically for intellectual content. ML wrote the first draft of the manuscript. All authors contributed to the article and approved the submitted version.

## Funding

This work was supported by Portuguese national funds provided by FCT—Fundação para a Ciência e Tecnologia, I.P., (UI/05704/2020), by the Portuguese Society of Diabetology through the award of the 2020 Emílio Peres Scholarship, and by the Faculty of Nutrition and Food Sciences of the University of Porto and LabbITR (LA/P/0064/2020). ML was supported by a PhD Scholarship from Fundação para a Ciência e Tecnologia (2021.07673.BD). MG was funded by national funds through FCT—Fundação para a Ciência e a Tecnologia, I.P., under the Scientific Employment Stimulus—Institutional Call—CEECINST/00051/2018.

## Conflict of interest

The authors declare that the research was conducted in the absence of any commercial or financial relationships that could be construed as a potential conflict of interest.

## Publisher’s note

All claims expressed in this article are solely those of the authors and do not necessarily represent those of their affiliated organizations, or those of the publisher, the editors and the reviewers. Any product that may be evaluated in this article, or claim that may be made by its manufacturer, is not guaranteed or endorsed by the publisher.

## References

[1] World Health Organization. Obesity and overweight [internet]. WHO (2021). Available at: https://www.who.int/news-room/fact-sheets/detail/obesity-and-overweight (Accessed 24 February 2022).

[2] Piché ME Tchernof A Després JP. Obesity Phenotypes, Diabetes, and cardiovascular diseases. Circ Res. (2020) 126:1477–500. doi: 10.1161/CIRCRESAHA.120.316101, PMID: 32437302

[3] González-Muniesa P Mártinez-González MA Hu FB Després JP Matsuzawa Y Loos RJF. Obesity. Nat Rev Dis Primers. (2017) 3:1–18. doi: 10.1038/nrdp.2017.34 28617414

[4] Di Angelantonio E Bhupathiraju SN Wormser D Gao P Kaptoge S de Gonzalez AB. Body-mass index and all-cause mortality: individual-participant-data meta-analysis of 239 prospective studies in four continents. Lancet. (2016) 388:776–86. doi: 10.1016/S0140-6736(16)30175-1, PMID: 27423262 PMC4995441

[5] Nishida C Barba C Cavalli-Sforza T Cutter J Deurenberg P Darnton-Hill I. Appropriate body-mass index for Asian populations and its implications for policy and intervention strategies. Lancet. (2004) 363:157–63. doi: 10.1016/S0140-6736(03)15268-3 14726171

[6] Gurunathan U Myles PS. Limitations of body mass index as an obesity measure of perioperative risk. Br J Anaesth. (2016) 116:319–21. doi: 10.1093/bja/aev541, PMID: 26865129

[7] Cornier M-A Després J-P Davis N Grossniklaus DA Klein S Lamarche B. Assessing adiposity. Circulation. (2011) 124:1996–2019. doi: 10.1161/CIR.0b013e318233bc6a, PMID: 21947291

[8] Cunha-Guimaraes JP Guarino MP Timóteo AT Caires I Sacramento JF Ribeiro MJ. Carotid body chemosensitivity: early biomarker of dysmetabolism in humans. Eur J Endocrinol. (2020) 182:549–57. doi: 10.1530/EJE-19-0976, PMID: 32213652

[9] Fonseca-Pinto R Lopes NV Brito GC Lages M Guarino MP. Assessing autonomic control of metabolic syndrome by principal component analysis: a data driven methodology. Health Technol. (2020) 10:79–85. doi: 10.1007/s12553-019-00384-7

[10] Vecchié A Dallegri F Carbone F Bonaventura A Liberale L Portincasa P. Obesity phenotypes and their paradoxical association with cardiovascular diseases. Eur J Intern Med. (2018) 48:6–17. doi: 10.1016/j.ejim.2017.10.020, PMID: 29100895

[11] Tsigos C Hainer V Basdevant A Finer N Fried M Mathus-Vliegen E. Management of Obesity in adults: European clinical practice guidelines. Obes Facts. (2008) 1:106–16. doi: 10.1159/000126822, PMID: 20054170 PMC6452117

[12] Lau DCW Douketis JD Morrison KM Hramiak IM Sharma AM Ur E. 2006 Canadian clinical practice guidelines on the management and prevention of obesity in adults and children [summary]. Can Med Assoc J. (2007) 176:S1–S13. doi: 10.1503/cmaj.061409, PMID: 17420481 PMC1839777

[13] MacLean PS Wing RR Davidson T Epstein L Goodpaster B Hall KD. NIH working group report: innovative research to improve maintenance of weight loss. Obesity. (2015) 23:7–15. doi: 10.1002/oby.20967, PMID: 25469998 PMC5841916

[14] Després JP. Body fat distribution and risk of cardiovascular disease: An update. Circulation. (2012) 126:1301–13. doi: 10.1161/CIRCULATIONAHA.111.067264, PMID: 22949540

[15] Smith GI Mittendorfer B Klein S. Metabolically healthy obesity: facts and fantasies. J Clin Invest. (2019) 129:3978–89. doi: 10.1172/JCI129186, PMID: 31524630 PMC6763224

[16] Després J-P Lemieux I Bergeron J Pibarot P Mathieu P Larose E. Abdominal Obesity and the metabolic syndrome: contribution to global Cardiometabolic risk. Arterioscler Thromb Vasc Biol. (2008) 28:1039–49. doi: 10.1161/ATVBAHA.107.159228, PMID: 18356555

[17] Laermans J Depoortere I. Chronobesity: role of the circadian system in the obesity epidemic. Obes Rev. (2016) 17:108–25. doi: 10.1111/obr.12351, PMID: 26693661

[18] Froy O. Metabolism and circadian rhythms—implications for obesity. Endocr Rev. (2010) 31:1–24. doi: 10.1210/er.2009-0014, PMID: 19854863

[19] Pan A Schernhammer ES Sun Q Hu FB. Rotating night shift work and risk of type 2 diabetes: two prospective cohort studies in women. PLoS Med. (2011) 8:e1001141. doi: 10.1371/journal.pmed.1001141, PMID: 22162955 PMC3232220

[20] Challet E. Keeping circadian time with hormones. Diabetes Obes Metab. (2015) 17:76–83. doi: 10.1111/dom.12516, PMID: 26332971

[21] Arble DM Ramsey KM Bass J Turek FW. Circadian disruption and metabolic disease: findings from animal models. Best Pract Res Clin Endocrinol Metab. (2010) 24:785–800. doi: 10.1016/j.beem.2010.08.003, PMID: 21112026 PMC3011935

[22] Morris CJ Purvis TE Hu K Scheer FAJL. Circadian misalignment increases cardiovascular disease risk factors in humans. Proc Natl Acad Sci U S A. (2016) 113:E1402–11. doi: 10.1073/pnas.1516953113, PMID: 26858430 PMC4790999

[23] Tahara Y Shibata S. Chronobiology and nutrition. Neuroscience. (2013) 253:78–88. doi: 10.1016/j.neuroscience.2013.08.049, PMID: 24007937

[24] Flanagan A Bechtold DA Pot GK Johnston JD. Chrono-nutrition: from molecular and neuronal mechanisms to human epidemiology and timed feeding patterns. J Neurochem. (2021) 157:53–72. doi: 10.1111/jnc.15246, PMID: 33222161

[25] Hirao A Tahara Y Kimura I Shibata S. A balanced diet is necessary for proper entrainment signals of the mouse liver clock. Yamazaki S, editor. PLoS One (2009);:e6909. doi: 10.1371/journal.pone.0006909, PMID: 19738906 PMC2734168

[26] Oike H Oishi K Kobori M. Nutrients, clock genes, and Chrononutrition. Curr Nutr Rep. (2014) 3:204–12. doi: 10.1007/s13668-014-0082-6, PMID: 25101217 PMC4118017

[27] Oike H Nagai K Fukushima T Ishida N Kobori M. Feeding cues and injected nutrients induce acute expression of multiple clock genes in the mouse liver. Yamazaki S, editor. PLoS One. (2011);6:e23709. doi: 10.1371/journal.pone.0023709, PMID: 21901130 PMC3162004

[28] Tahara Y Otsuka M Fuse Y Hirao A Shibata S. Refeeding after fasting elicits insulin-dependent regulation of Per2 and rev-erbα with shifts in the liver clock. J Biol Rhythm. (2011) 26:230–40. doi: 10.1177/0748730411405958, PMID: 21628550

[29] Vollmers C Gill S DiTacchio L Pulivarthy SR Le HD Panda S. Time of feeding and the intrinsic circadian clock drive rhythms in hepatic gene expression. Proc Natl Acad Sci. (2009) 106:21453–8. doi: 10.1073/pnas.0909591106, PMID: 19940241 PMC2795502

[30] Itokawa M Hirao A Nagahama H Otsuka M Ohtsu T Furutani N. Time-restricted feeding of rapidly digested starches causes stronger entrainment of the liver clock in PER2::LUCIFERASE knock-in mice. Nutr Res. (2013) 33:109–19. doi: 10.1016/j.nutres.2012.12.004, PMID: 23399661

[31] Henry CJ Kaur B Quek RYC. Chrononutrition in the management of diabetes. Nutr Diabetes. (2020) 10:6. doi: 10.1038/s41387-020-0109-6 32075959 PMC7031264

[32] Fuse Y Hirao A Kuroda H Otsuka M Tahara Y Shibata S. Differential roles of breakfast only (one meal per day) and a bigger breakfast with a small dinner (two meals per day) in mice fed a high-fat diet with regard to induced obesity and lipid metabolism. J Circadian Rhythms. (2012) 10:4. doi: 10.1186/1740-3391-10-4/, PMID: 22587351 PMC3434038

[33] Wu T Sun L ZhuGe F Guo X Zhao Z Tang R. Differential roles of breakfast and supper in rats of a daily three-meal schedule upon circadian regulation and physiology. Chronobiol Int. (2011) 28:890–903. doi: 10.3109/07420528.2011.622599, PMID: 22080734

[34] Jakubowicz D Wainstein J Ahrén B Bar-Dayan Y Landau Z Rabinovitz HR. High-energy breakfast with low-energy dinner decreases overall daily hyperglycaemia in type 2 diabetic patients: a randomised clinical trial. Diabetologia. (2015) 58:912–9. doi: 10.1007/s00125-015-3524-9, PMID: 25724569

[35] Jakubowicz D Wainstein J Landau Z Raz I Ahren B Chapnik N. Influences of breakfast on clock gene expression and postprandial Glycemia in healthy individuals and individuals with diabetes: a randomized clinical trial. Diabetes Care. (2017) 40:1573–9. doi: 10.2337/dc16-2753, PMID: 28830875

[36] Galindo Muñoz JS Gómez Gallego M Díaz Soler I Barberá Ortega MC Martínez Cáceres CM Hernández Morante JJ. Effect of a chronotype-adjusted diet on weight loss effectiveness: a randomized clinical trial. Clin Nutr. (2020) 39:1041–8. doi: 10.1016/j.clnu.2019.05.012, PMID: 31153674

[37] Hawley JA Sassone-Corsi P Zierath JR. Chrono-nutrition for the prevention and treatment of obesity and type 2 diabetes: from mice to men. Diabetologia. (2020) 63:2253–9. doi: 10.1007/s00125-020-05238-w, PMID: 32761356

[38] Lévi F Okyar A Dulong S Innominato PF Clairambault J. Circadian timing in cancer treatments. Annu Rev Pharmacol Toxicol. (2010) 50:377–421. doi: 10.1146/annurev.pharmtox.48.113006.094626, PMID: 20055686

[39] Evans JA Davidson AJ. Health consequences of circadian disruption in humans and animal models In: Progress in molecular biology and translational science. Gillette M. U. (Cambridge, Massachusetts: Elsevier B.V) (2013). 283–323. 10.1016/B978-0-12-396971-2.00010-5 23899601

[40] Gaspar LS Álvaro AR Carmo-Silva S Mendes AF Relógio A Cavadas C. The importance of determining circadian parameters in pharmacological studies. Br J Pharmacol. (2019) 176:2827–47. doi: 10.1111/bph.14712, PMID: 31099023 PMC6637036

[41] Roenneberg T Wirz-Justice A Merrow M. Life between clocks: daily temporal patterns of human Chronotypes. J Biol Rhythm. (2003) 18:80–90. doi: 10.1177/0748730402239679, PMID: 12568247

[42] Moon S Kang J Kim SH Chung HS Kim YJ Yu JM. Beneficial effects of time-restricted eating on metabolic diseases: a systemic review and meta-analysis. Nutrients. (2020) 12:1267. doi: 10.3390/nu12051267, PMID: 32365676 PMC7284632

[43] Dashti HS Gómez-Abellán P Qian J Esteban A Morales E Scheer FAJL. Late eating is associated with cardiometabolic risk traits, obesogenic behaviors, and impaired weight loss. Am J Clin Nutr. (2021) 113:154–61. doi: 10.1093/ajcn/nqaa264, PMID: 33022698 PMC7779221

[44] Adafer R Messaadi W Meddahi M Patey A Haderbache A Bayen S. Food timing, circadian rhythm and Chrononutrition: a systematic review of time-restricted Eating’s effects on human health. Nutrients. (2020) 12:3770. doi: 10.3390/nu12123770, PMID: 33302500 PMC7763532

[45] Chan A-W Tetzlaff JM Altman DG Laupacis A Gøtzsche PC Krleža-Jerić K. SPIRIT 2013 statement: defining standard protocol items for clinical trials. Ann Intern Med. (2013) 158:200. doi: 10.7326/0003-4819-158-3-201302050-00583, PMID: 23295957 PMC5114123

[46] Bland M. An introduction to medical statistics. 4th ed Oxford, United Kingdom: Oxford University Press (2015).

[47] World Obesity. (2022). Global obesity observatory. Available at: https://data.worldobesity.org/country/portugal-174/ (Accessed 30 December 2022).

[48] Loos RJF Yeo GSH. The genetics of obesity: from discovery to biology. Nat Rev Genet. (2021) 23:120–33. doi: 10.1038/s41576-021-00414-z 34556834 PMC8459824

[49] Direção Geral de Saúde. Avaliação Antropométrica no Adulto – Orientação da DGS n.o 017/2013 de 05 de Dezembro. (2013). Available at:: https://www.dgs.pt/directrizes-da-dgs/orientacoes-e-circulares-informativas/orientacao-n-0172013-de-05122013-pdf.aspx

[50] World Health Organization. WHO/Europe | nutrition—body mass index—BMI. (2021) Available at: http://www.euro.who.int/en/health-topics/disease-prevention/nutrition/a-healthy-lifestyle/body-mass-index-bmi (Accessed 24 February 2022).

[51] Gaspar LS Hesse J Yalçin M Santos B Carvalhas-Almeida C Ferreira M. Long-term continuous positive airway pressure treatment ameliorates biological clock disruptions in obstructive sleep apnea. EBioMedicine. (2021) 65:103248. doi: 10.1016/j.ebiom.2021.103248, PMID: 33647771 PMC7920825

[52] Livak KJ Schmittgen TD. Analysis of relative gene expression data using real-time quantitative PCR and the 2−ΔΔCT method. Methods. (2001) 25:402–8. doi: 10.1006/meth.2001.1262, PMID: 11846609

[53] Rosa I Lages M Grilo C Barros R Guarino MP. mHealth applications to monitor lifestyle behaviors and circadian rhythm in clinical settings: current perspective and future directions. Front Public Health. (2022) 10:862065. doi: 10.3389/fpubh.2022.862065, PMID: 35923965 PMC9339674

[54] National Institute of health Doutor Ricardo Jorge. Food Composition Table (2021). Available at: http://portfir.insa.pt/ (Accessed 2 December 2022).

[55] Food E Authority S Ambrus A Busk L Bush M Finglas P. Guidance on the EU menu methodology. EFSA J. (2014) 12:3944. doi: 10.2903/j.efsa.2014.3944

[56] Lopes C Aro A Azevedo A Ramos E Barros H. Intake and adipose tissue composition of fatty acids and risk of myocardial infarction in a male Portuguese community sample. J Am Diet Assoc. (2007) 107:276–86. doi: 10.1016/j.jada.2006.11.008, PMID: 17258965

[57] Gomes AA Marques DR Meiavia AM Cunha F Clemente V. Psychometric properties and accuracy of the European Portuguese version of the Pittsburgh sleep quality index in clinical and non-clinical samples. Sleep Biol Rhythms. (2018) 16:413–22. doi: 10.1007/s41105-018-0171-9

[58] Buysse DJ Reynolds CF Monk TH Berman SR Kupfer DJ. The Pittsburgh sleep quality index: a new instrument for psychiatric practice and research. Psychiatry Res. (1989) 28:193–213. doi: 10.1016/0165-1781(89)90047-4, PMID: 2748771

[59] Del Rio João KA Becker NB de Neves JS Martins IS. Validation of the Portuguese version of the Pittsburgh sleep quality index (PSQI-PT). Psychiatry Res. (2017) 247:225–9. doi: 10.1016/j.psychres.2016.11.042, PMID: 27923147

[60] Roenneberg T Pilz LK Zerbini G Winnebeck EC. Chronotype and social jetlag: a (self-). Crit Rev Biol. (2019) 8:54. doi: 10.3390/biology8030054 PMC6784249 31336976

[61] Terman JS Terman M Lo E-S Cooper TB. Circadian time of morning light administration and therapeutic response in winter depression. Arch Gen Psychiatry. (2001) 58:69–75. doi: 10.1001/archpsyc.58.1.69, PMID: 11146760

[62] Reis C Madeira SG Lopes LV Paiva T Roenneberg T. Validation of the Portuguese variant of the Munich Chronotype questionnaire (MCTQPT). Front Physiol. (2020) 11:795. doi: 10.3389/fphys.2020.00795/full, PMID: 32760292 PMC7372122

[63] Gamble KL Berry R Frank SJ Young ME. Circadian clock control of endocrine factors. Nat Rev Endocrinol. (2014) 10:466–75. doi: 10.1038/nrendo.2014.78, PMID: 24863387 PMC4304769

[64] Teoh AN Kaur S Shafie SR Mohd Shukri NH Ahmad Bustami N Takahashi M. Chrononutrition is associated with melatonin and cortisol rhythm during pregnancy: findings from MY-CARE cohort study. Front Nutr. (2023) 9:3221. doi: 10.3389/fnut.2022.1078086 PMC9852999 36687684

[65] Morris CJ Yang JN Garcia JI Myers S Bozzi I Wang W. Endogenous circadian system and circadian misalignment impact glucose tolerance via separate mechanisms in humans. Proc Natl Acad Sci U S A. (2015) 112:E2225–34. doi: 10.1073/pnas.1418955112, PMID: 25870289 PMC4418873

[66] Scheer FAJL Morris CJ Garcia JI Smales C Kelly EE Marks J. Repeated melatonin supplementation improves sleep in hypertensive patients treated with Beta-blockers: a randomized controlled trial. Sleep. (2012) 35:1395–402. doi: 10.5665/sleep.2122, PMID: 23024438 PMC3443766

[67] Stenvers DJ Scheer FAJL Schrauwen P la Fleur SE Kalsbeek A. Circadian clocks and insulin resistance. Nat Rev Endocrinol. (2019) 15:75–89. doi: 10.1038/s41574-018-0122-1, PMID: 30531917

[68] Cipolla-Neto J Amaral FG Afeche SC Tan DX Reiter RJ. Melatonin, energy metabolism, and obesity: a review. J Pineal Res. (2014) 56:371–81. doi: 10.1111/jpi.12137, PMID: 24654916

[69] Genario R Cipolla-Neto J Bueno AA Santos HO. Melatonin supplementation in the management of obesity and obesity-associated disorders: a review of physiological mechanisms and clinical applications. Pharmacol Res. (2021) 163:105254. doi: 10.1016/j.phrs.2020.105254, PMID: 33080320

[70] Guan Q Wang Z Cao J Dong Y Chen Y. Mechanisms of melatonin in obesity: a review. Int J Mol Sci. (2021) 23:–218. doi: 10.3390/ijms23010218, PMID: 35008644 PMC8745381

[71] Ruddick-Collins LC Morgan PJ Johnstone AM. Mealtime: a circadian disruptor and determinant of energy balance? J Neuroendocrinol. (2020) 32:e12886. doi: 10.1111/jne.12886, PMID: 32662577

[72] Kim YH Lazar MA. Transcriptional control of circadian rhythms and metabolism: a matter of time and space. Endocr Rev. (2020) 41:707–32. doi: 10.1210/endrev/bnaa014, PMID: 32392281 PMC7334005

[73] Glicksman A Hadjiyannakis S Barrowman N Walker S Hoey L Katz SL. Body fat distribution ratios and obstructive sleep apnea severity in youth with obesity. J Clin Sleep Med. (2017) 13:545–50. doi: 10.5664/jcsm.6538, PMID: 28095969 PMC5359330

[74] Benjafield AV Ayas NT Eastwood PR Heinzer R Ip MSM Morrell MJ. Estimation of the global prevalence and burden of obstructive sleep apnoea: a literature-based analysis. Lancet Respir Med. (2019) 7:687–98. doi: 10.1016/S2213-2600(19)30198-5, PMID: 31300334 PMC7007763

[75] Asher G Sassone-Corsi P. Time for Food: the intimate interplay between nutrition, metabolism, and the circadian clock. Cells. (2015) 161:84–92. doi: 10.1016/j.cell.2015.03.015, PMID: 25815987

[76] Garaulet M Corbalán-Tutau MD Madrid JA Baraza JC Parnell LD Lee Y-C. PERIOD2 variants are associated with abdominal obesity, psycho-behavioral factors, and attrition in the dietary treatment of obesity. J Am Diet Assoc. (2010) 110:917–21. doi: 10.1016/j.jada.2010.03.017, PMID: 20497782 PMC4428932

[77] Garaulet M Gómez-Abellán P Alburquerque-Béjar JJ Lee Y-C Ordovás JM Scheer FAJL. Timing of food intake predicts weight loss effectiveness. Int J Obes. (2013) 37:604–11. doi: 10.1038/ijo.2012.229, PMID: 23357955 PMC3756673

[78] Dupuis J Langenberg C Prokopenko I Saxena R Soranzo N Jackson AU. New genetic loci implicated in fasting glucose homeostasis and their impact on type 2 diabetes risk. Nat Genet. (2010) 42:105–16. doi: 10.1038/ng.520, PMID: 20081858 PMC3018764

[79] Jamshed H Beyl R Della Manna D Yang E Ravussin E Peterson C. Early time-restricted feeding improves 24-hour glucose levels and affects markers of the circadian clock, aging, and autophagy in humans. Nutrients. (2019) 11:1234. doi: 10.3390/nu11061234, PMID: 31151228 PMC6627766

[80] Sharp DB Allman-Farinelli M. Feasibility and validity of mobile phones to assess dietary intake. Nutrition. (2014) 30:1257–66. doi: 10.1016/j.nut.2014.02.020, PMID: 24976425

[81] Goris AHC Westerterp-Plantenga MS Westerterp KR. Undereating and underrecording of habitual food intake in obese men: selective underreporting of fat intake. Am J Clin Nutr. (2000) 71:130–4. doi: 10.1093/ajcn/71.1.130, PMID: 10617957

[82] Hebert JR Hurley TG Peterson KE Resnicow K Thompson FE Yaroch AL. Social desirability trait influences on self-reported dietary measures among diverse participants in a multicenter multiple risk factor trial. J Nutr. (2008) 138:226S–34S. doi: 10.1093/jn/138.1.226S, PMID: 18156429

